# Refractory psychiatric symptoms and seizure associated with Dandy-Walker syndrome: A case report and literature review

**DOI:** 10.1097/MD.0000000000031421

**Published:** 2022-11-18

**Authors:** Yijing Chen, Junhong Zhu, Di Zhang, Li Han, Juan Wang, Weiwei Yang

**Affiliations:** a Wuhan Mental Health Center, Wuhan, China; b Wuhan Hospital for Psychotherapy, Wuhan, China.

**Keywords:** case report, Dandy-Walker syndrome, hydrocephalus, psychosis, schizophrenia, seizure, ventriculoperitoneal shunting

## Abstract

Dandy-Walker syndrome (DWS) is a group of brain malformations which occasionally accompanied by psychotic symptoms. The co-occurrence of DWS and epilepsy in children is quite rare.

**Case description:**

We reported a 14-year-old male who presented with a 8-month history of inconsistent upper limb tremor and accidental seizure. The MRI showed the typical alterations of DWS: cystic dilatation of the fourth ventricle, vermian hypoplasia, enlarged posterior fossa. He received the ventriculoperitoneal shunting (VPS) placement for hydrocephalus and had a symptom-free period for 8 days. Then he experienced a recurrence of involuntary upper limb tremor and behavior disturbance after decreasing the pressure of cerebrospinal fluid (CSF) from 150 to 130 mm Hg. After being treated with Olanzapine 10 mg/d, Clonazepam 3 mg/qn and Valproate acid (VPA) 500 mg/bid for nearly a month, his mental status and psychotic symptoms fluctuated. A search of Pub Med showed little report of hydrocephalus and DWS comorbidity with seizure and psychosis. Here we presented the whole process of a rare disease from the very beginning with all his symptoms, examinations and treatments.

**Conclusion:**

VPS placement surgery at an earlier stage may be an effective way to avoid inevitable brain damage so as to improve the clinical outcomes for patients with DWS. Continued treatment with regard to DWS condition may include shunt placement, but it mainly focus on developmental concerns, with occupational and physical therapy along with ongoing supportive psychotherapy to improve the coping skills and quality of life.

## 1. Introduction

DWM is a rare congenital neurological malformation typically characterized as cystic dilatation of the fourth ventricle, vermian hypoplasia, posterior fossa cyst and hydrocephalus.^[1]^ The main etiological factors are assumed to be altered nucleic acid synthesis, chromosomal aberrations and mutations as a consequence of the deficiency in granule cell precursors expansion.^[2–4]^ Congenital or acquired cerebellar lesions are reported to be associated with psychiatric disorders.^[5]^ The cerebellar structures, especially the vermis, are connected with the psychopathology of schizophrenia and related psychiatric symptoms, such as cognitive dysfunctions and auditory verbal hallucinations.^[2,6]^ The cerebellar are also responsible for the neurodevelopmental complications in DWM and nearly 80% to 90% of children present abnormality in the first year of their life, common symptoms are cranial and cerebellar nerves dysfunction, hydrocephalus and the presence of related anomalies, such as macrocrania, delayed motor skill and cognitive development.^[1,7]^ Some patients may present as seizure, which is usually connected with supranational malformations and accompanied by audio-visual problems and together with insufficient intelligence development.^[8]^ Hydrocephalus is common in DWM and presents in nearly 80% of the cases.^[7]^ The occurrence of hydrocephalus and ventriculomegaly requiring a VPS to reduce raised intracranial pressure (ICP) occurred in 62.7% of cases.^[9]^ The treatment of Dandy-Walker syndrome-related hydrocephalus requires either endoscopic third ventriculostomy-based or a CSF shunt-based procedure.^[10]^ Commonly employed CSF surgeries were VPS and ventriculopleural shunt.^[11]^ There has been a considerable decrease in morbidity and mortality rates since the use of shunts.^[1]^ However, structural brain damage, shunt revision and complications are independent high risk factors for developing seizure in children with hydrocephalus.^[12]^

Reviewing the literature, the authors have found a few published cases describing co-occurrence of psychotic symptoms and DWS.^[2,5,13–16]^ However, few report has described the patient with DWM and hydrocephalus after undergoing surgery. In the present study, we describe a case of a young man who was diagnosed with DWM and seizure co-morbid with suspected atypical schizophrenia-like symptoms. This case is to our knowledge the first to describe DWM and hydrocephalus along with a new-onset seizure treated with VPS insertion, which experienced a symptom-free interval and then a recurrence of psychosis right after the adjustment of CSF pressure. Schizophrenia-like symptoms induced by neurological lesions may present clinical features that differentiate them from primary schizophrenia, which may result in the need for different treatment approaches.^[13]^ The case may further illuminate the physio-pathology and treatment strategy of psychotic disorders with congenital cerebellum malformation.

## 2. Case description

A 14-year-old male diagnosed with an 8-month history of psychosis, presented as schizophrenia-like symptoms, including emotionally unstable, auditory hallucinations, involuntary movement of the head, suspected delusions, repetitive peculiar behavior and inconsistent convulsion on the upper arm. He had multiple episodes of tonic-clonic seizure in a day and lasted for around 20 seconds each time. He presented manic episode and violent behavior occasionally. He also had a quite unstable mood and could shifted from extremely aggressive to totally peace in seconds. He sang the same song and walked in the ward back and forth repetitively. The psychological CT revealed a severe abnormality in mental state in November 2020. Treatment with Olanzapine 2.5 mg/d, Valproic Acid 500 mg/d, and Artane 2 mg/d were given in another psychiatric hospital, and the symptoms remained controlled within the following 6.5 months until his next visit to the clinic, where he had a tonic-clonic seizure attack on both of his upper arms and was admitted to the hospital for treatment. Re-administration of the above-mentioned treatment with a maximum of Olanzapine 2.5 mg/d did not relieve his symptoms this time, he had restless and emotionally unstable. He was transferred to another hospital where he was diagnosed as DWS and hydrocephalus. The laboratory testing of CSF showed no abnormality (CSF protein test: negative; CSF bacteria culture: negative; autoimmune antibody: negative). The total protein level 0.3 g/L, and Glucose 3.99 mmol/L. Pr-surgery MRI + Diffusion Weighted Imaging showed enlarged cisterna magna and ventricle system. The abdominal CT showed a pelvic effusion. He underwent the VPS placement for the management of hydrocephalus. During the next 7 days, the patients experienced a seizure-free or psychotic symptoms-free along with a normal mental status period. However, when the CSF pressure was adjusted from 150 to 130 mm Hg by the physician, all his above mentioned symptoms recurred at the next day, he experienced the seizure attack (neurological examination: drowsiness; Glasgow Coma Scale: E4V4M5). The post-surgery MRI disclosed a DWM with hypoplasia of the cerebellar vermis, cystic dilatation of the fourth ventricle, enlarged posterior fossa and hydrocephalus; drainage tube image in the right ventricle. Then he was transferred to the mental health center and diagnosed with epilepsy, intellectual disability and organic delusional (schizophrenia-like) disorder on the basis of International Classification of Diseases, 10th edition criteria.

**Figure 1.**
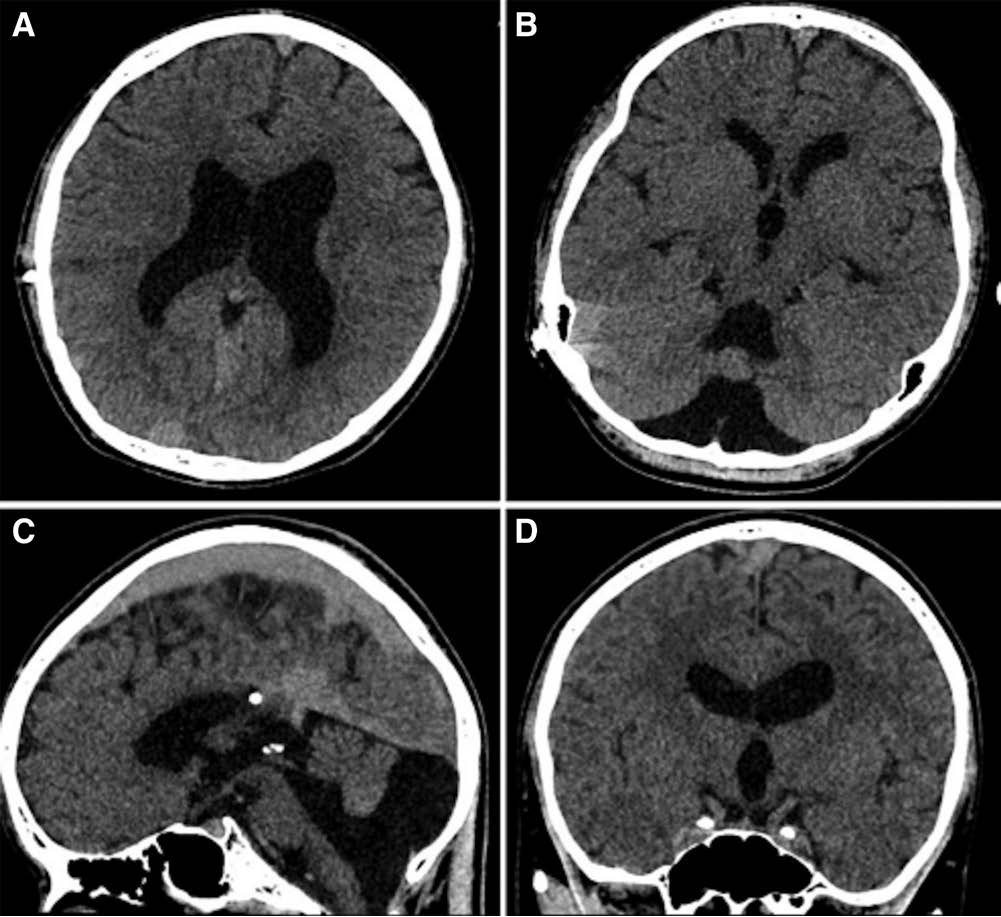
MR images of the patient (July 8, 2021). (a) An enlarged bilateral ventricle. (b/c) Hypoplasia of the cerebellar vermis, cystic dilatation of the fourth ventricle, enlarged posterior fossa with upward displacement of sinus confluens. (d) Dilatation of the cerebral ventricle and the third ventricle. MRI = magnetic resonance imaging.

Physical and clinical examinations were unremarkable but had an abnormal gait. Wechsler Intelligence Scale for Children confirmed mild intellectual disability (IQ-65). Blood gas analysis showed the results of PCO_2_ 8 Kpa, Lac 1.7 mmol/L, Ca^2 + ^1.10 mmol/L, Na^ + ^134 mmol/L, Glucose 6.8 mmol/L, AB 27.2 mmol/L, SB 26.8 mmol/L, SaO_2_ 91% and PaO_2_/FiO_2_ = 286. No remarkable abnormality was showed in laboratory tests, including the blood coagulation, inflammatory markers, EEG (Fig. 1) and ECG.

**Figure 2.**
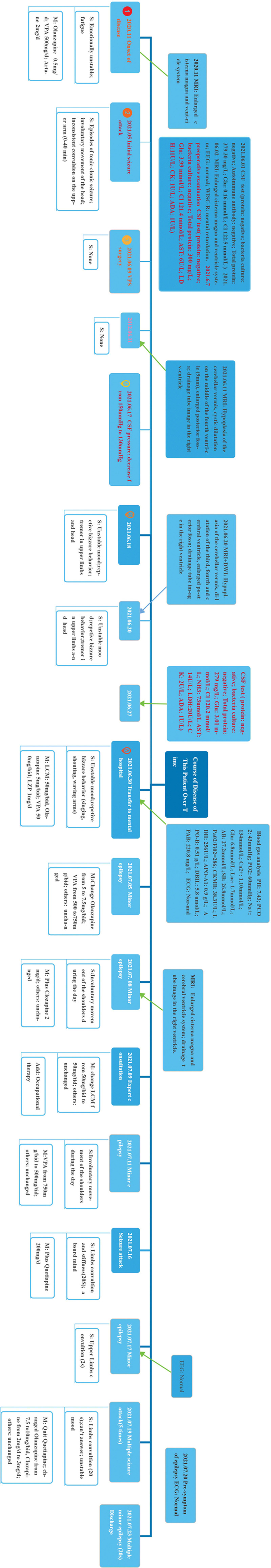
Course of treatment of this patient over time.

Another brain MRI taken on July 8 showed a similar result (Fig. S1, http://links.lww.com/MD/H772). His mother’s physical condition was reported as normal during pregnancy. He was delivered by cesarean section and achieved normal developmental milestones. Neither family history of psychosis nor substance abuse was reported. He was distracted and showed a decreased emotional reaction during mental status examination. He did not cooperate with memory and intelligence check. He was initially treated with VPA 500 mg/bid and Olanzapine 5 mg/bid, which were increased to VPA 500 mg/tid and Olanzapine 10 mg/bid gradually, plus Lacosamide 50mg/tid, Clozapine 3 mg/qn. Besides, he received occupational therapy 5 times per week. After 3 weeks of treatment, his score in BPRS varied from 19 to 30, then dropped to 19. The treatment response was inadequate, the symptoms of seizures, unstable mood and repetitive bizarre behaviors still persisted. The whole course of the disease was showed in Figure 2.

The study protocol was approved by the ethics committee of Wuhan Mental Health Center. The patient gave informed consent for the publication of this case.

## 3. Discussion

DWM is a rare congenital hypoplasia of the cerebellum often coexist with agenesis of corpus callosum, hydrocephalus and other intracranial malformation.^[17]^ Hydrocephalus is common in DWM, and increased ICP lead to additional symptoms, which may manifest with intellectual disability and seizures.^[14]^ The goal of continued treatment with regard to his DWM in the control of posterior fossa cyst and hydrocephalus required shunt placement.^[1]^ Nearly 30% of children with VPS placement have recurrent seizure.^[12]^ The patient described in this report, who had received VPS placement after being diagnosed with DWM and hydrocephalus, experienced a recurrence of behavioral and emotional disturbance after the adjustment of CSF pressure. Determining causality in this case is complicated by the number of confounding factors that may contribute to the recurrence of seizures and mental disturbance in this patient. In our case, 3 mechanisms might contribute to the recurrence of tremor, epilepsy and behavior disturbance: hydrocephalus and DWS related cortical damage, VPS surgery-related complication and certain anti-psychotics medication. Our case presented with obvious hypoplasia of the cerebellar vermis, cystic dilatation of the fourth ventricle before and after the surgery, which probably contributed to the disturbance of periphery and cerebellar feedback system.^[17]^ Patients with pathological focal lesion and cortical dysplasia had lower seizure threshold.^[18]^ Hydrocephalus can cause neuronal damage in the cortex by disturbing activity of the dopaminergic and noradrenergic neuronal system as well as to synaptogenesis, in advanced hydrocephalus, morphological deformation of cortical neurons are accompanied by variation in neurofilament immunoreactivity, since the neurofilament is the key component of the neuronal cytoskeleton which takes part in the maintenance of cytoplasmic morphology, functional and structural changes to the cytoskeleton can affect cortical neurons because of the procession of hydrocephalus, moreover, the axonal lesion in the periventricular white matter presented with the most incomplete recovery after shunt placement could attribute to the impaired cognitive function who have received shunt placement and have obviously restructured cerebral mantles.^[19]^

Shunt-related seizure is reported in nearly 48% of the patients and are suggested to be connected with the insertion of the VPS, seizure can be observed after VPS in children who have not presented any sign of hemorrhage or infection as underlying causes, the occurrence of shunt-related seizure might be explained by the reparation of the brain tissue and the injury to the brain parenchyma through ventricular catheter or the immunologic reaction to the catheter itself.^[20]^ What’s more, shunt malformation can provoke seizures and behavioral changes,^[21,22]^ the intracranial hemorrhage is associated with subsequent neurological and psychiatric symptoms.^[23]^ Alteration in cerebellum have been widely connected with epilepsy, including varied perfusion, changes in volume, disrupted structural and functional connectivity.^[24]^ Previous reports have described patients with shunt over-drainage-related refractory epilepsy and psychosis-like symptoms associated with increased ICP.^[21,25]^ One study report an acute cerebral hemorrhage after adjusting the valve pressure of a VPS placement, a sudden switch from 70 to 40 mmH_2_O triggered the fatal intracerebral bleeding possibility by tearing or a transient occlusion of the vein-bridge.^[26]^ Another study show a patients who received craniectomy for traumatic intracranial hemorrhage and had no obvious symptoms of seizure or sequela for the next 8 years, had a sudden onset of delusion, hallucination and general tonic-clonic seizure, possible explanation was that the preexisting brain injury had reduced the threshold of neurological deficits.^[27]^ However, in this case, the MR scan and CSF imaging showed no obvious evidence of hemorrhage or shunt malformation. One possible explanation is that shunt placement might repair the brain tissue and restore the damage caused during ventricular distention, shunt-related seizure could be related to the reparation of the tissue which was not possible before VPS insertion. Besides, delayed mental development and seizure could be the cause of pr-existing brain damage and shunt placement-related injury, not shunt-related.^[28]^ Patients who develop mental disorder have disturbances of cognition and behavior before the onset of symptoms as well as marked delays in mental and motor milestones,^[14]^ and VPS plays a limited role in changing the pr-existing neurological damage.^[28]^ Moreover, children with hydrocephalus had a incidence of 40% for developing seizure,^[12]^ and the risk of epilepsy increased by 20% for patients received VPS placement.^[21]^ Both shunting and hydrocephalus can change the micro-structure of brain and thus increased brain stiffness, which may explain the influence of minor variation in ICP on the function of brain tissue and subsequent episode of seizure.^[21,29]^

Certain anti-psychotics medication can reduce the seizure threshold, such as Clozapine and Bupropion.^[30]^ Olanzapine shared a similar pharmacological profile with the Clozapine in structure,^[31]^ it can precipitate seizure in a dose-dependent manner with a reported hazard rate of 0.6% to 14%, and the risk of seizures accumulates with the usage of anti-psychotics in patients with traumatic brain injury, abnormal EEG, preexisting central nervous system complications and seizure disorder.^[32]^ In addition, drug metabolism and combination with other anti-psychotic drugs are risk factors for Olanzapine-induced seizures.^[33]^ The potential mechanism is that some anti-psychotic drugs can directly or indirectly modulate neurotransmitter release so as to affect the threshold for seizure activity.^[34]^ Cycloxygenase-2 (COX-2) which expressed mostly in brain and kidneys is proved to be associated with seizure suppression, under the pathophysiological condition of the brain, COX-dependent PG production accumulates transiently and rapidly in the brain following seizures and the inactivation of the COX-2 gene reduces the innate seizure threshold.^[35]^ Besides, enhanced mTOR activation can foster epileptogenesis, and it can be a critical activation step even without obvious epilepsy-associated pathology as well as distinct neuropathological changes.^[36]^

## 4. Conclusion

The current study shows a 14-year-old male patient with DWS and hydrocephalus, who also presents the symptoms of seizure and behavior disturbance. He underwent the VPS placement for the management of hydrocephalus and experienced a symptom-free period. The surgery may improve the clinical symptoms for patients with DWS temporarily, while some neurological damage is pr-existing and cannot be revised. Continued treatment with regard to DWS condition may include shunt placement, but it mainly focus on developmental concerns, with occupational and physical therapy along with ongoing supportive psychotherapy to improve the coping skills and quality of life.

## Author contributions

ZH and DZ involves in the conception and design of the work. YC wrote and revised the first draft of the manuscript. LH, JW and WY revised the manuscript and analyzing the clinical data. All authors contributed to the article and approved the submitted version.

**Conceptualization:** JH Zhu.

**Formal analysis:** YJ Chen.

**Investigation:** L Han.

**Project administration:** YJ Chen, JH Zhu, D Zhang, L Han.

**Resources:** L Han.

**Supervision:** D Zhang, L Han.

**Validation:** JH Zhu, D Zhang, WW Yang.

**Visualization:** WW Yang.

**Writing – original draft:** YJ Chen.

**Writing – review & editing:** JH Zhu, D Zhang, J Wang.

## Supplementary Material
