# Supplementary figures and images for: Refractory psychiatric symptoms and seizure associated with Dandy-Walker syndrome: A case report and literature review
Source: Medicine (Baltimore). 2022 Nov 18;101(46):e31421. doi: 10.1097/MD.0000000000031421 (PMC9678574; doi:10.1097/MD.0000000000031421)

Supplementary fig 1: Results of electroencephalography showed a normal wave

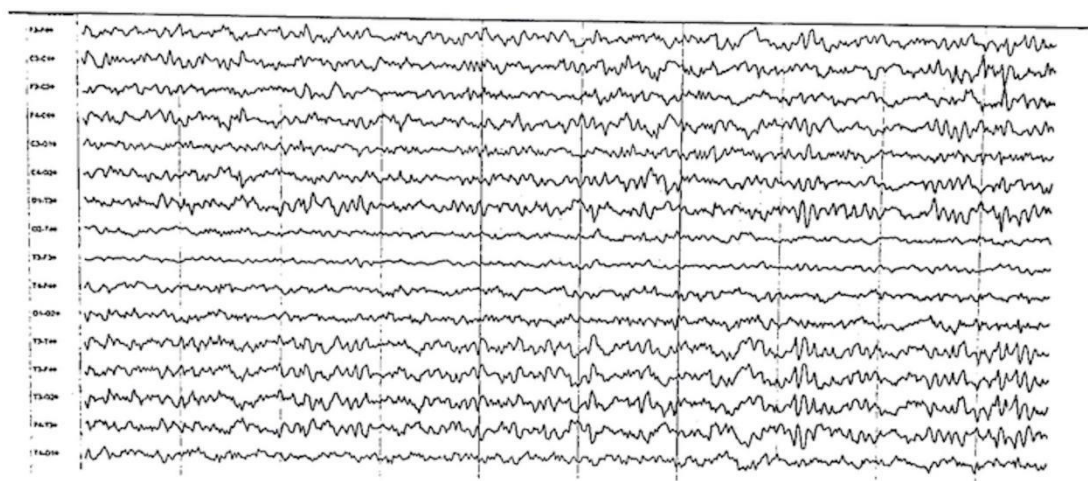

Supplement: Supplementary file 1 [file medi-101-e31421-s001.pdf]
